# Supplementary material for: A Patient-Ready Wearable Transcutaneous CO2 Sensor
Source: Biosensors (Basel). 2022 May 13;12(5):333. doi: 10.3390/bios12050333 (PMC9138690; doi:10.3390/bios12050333)
Supplement: Supplementary file 1 [file biosensors-12-00333-s001.zip › biosensors-1710480-supplementary.pdf]

Supplementary Material

A patient-ready wearable transcutaneous CO_2 sensor

Juan Pedro Cascales, Xiaolei Li, Emmanuel Rousakis, and Conor L. Evans*

Wellman Center for Photomedicine, Massachusetts General Hospital, Harvard Medical School, Charlestown, 02129, MA

E-mail: evans.conor@mgh.harvard.edu

Material aging and sensitivity

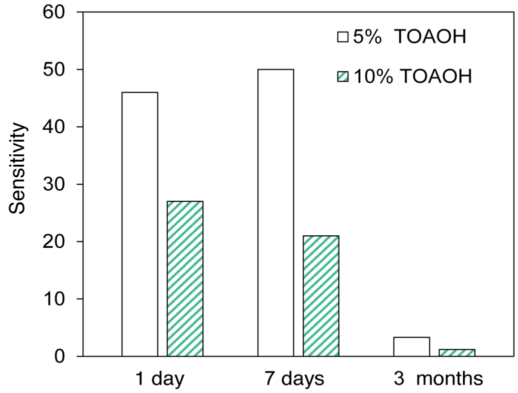

Figure S1: Effect of aging time on sensitivity under ambient, dark condition for materials made of (HPTS)/$(TOA)_4$ in PPMA with 5% and 10% TOAOH.

Materials spectral characterization

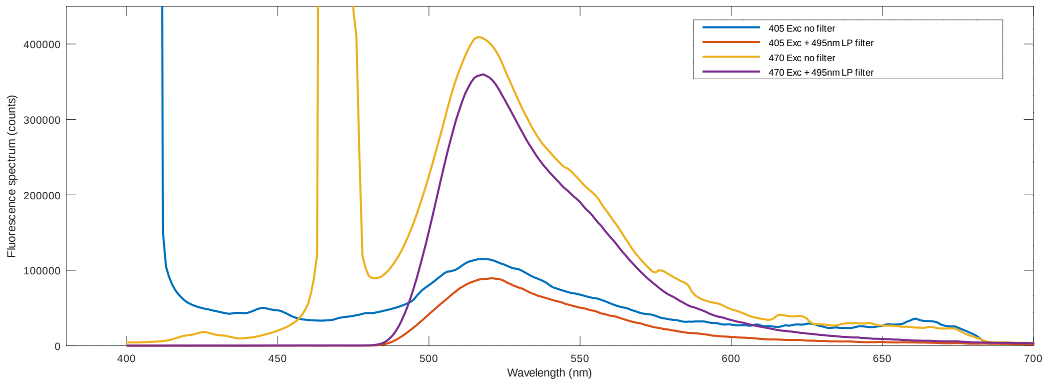

Figure S2: Spectra of HTPS(TOA)4/PPMA taken with the reference Edinburgh spectrometer, excited at 405 and 470nm, with and without the 495 longpass filter.

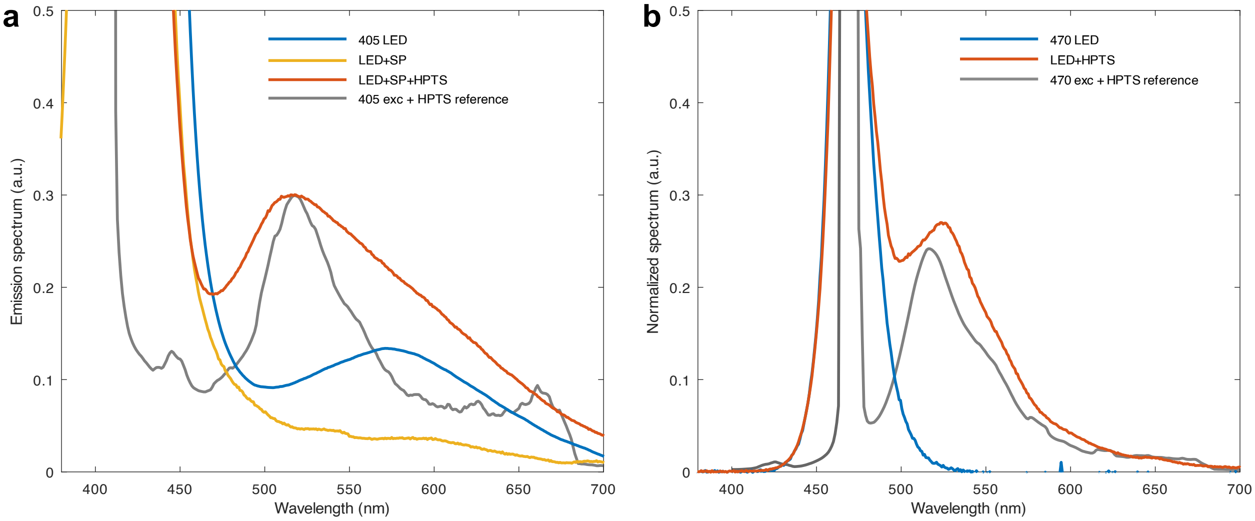

Figure S3: Spectra measured from the wearable device, compared to the spectra from the reference spectrometer from Fig.S2 (gray lines). (a) 405 nm LED shows an unwanted phosphorescence which is filtered out using short-pass, flexible filters (SP) which allow the HPTS emission to shine through. (b) The 470 nm LED does not require such filtering.

Calibration system

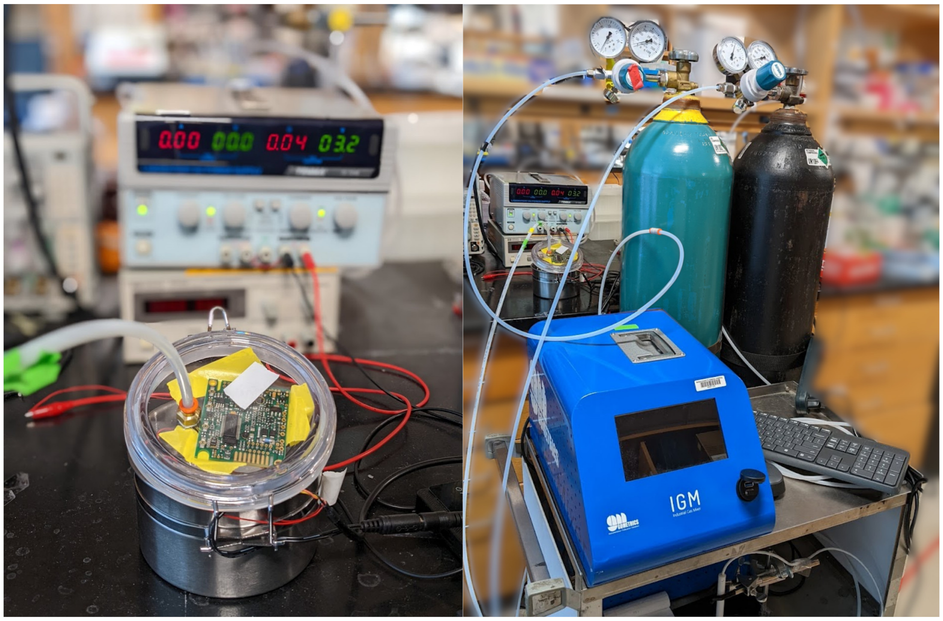

Figure S4: Calibration setup, including a sealed chamber with heater and reference CO_2 sensor, CO_2 and N_2 tanks and an automated gas mixer.

Fluorescence ratio R via integration of the spectrum

The definition of of $I_{405,470}$ as the integral of the fluorescence spectrum (with a long-pass filter to remove the excitation), and the ratio $R = I_{405}/I_{470}$ should be equivalent to the trends observed if $I_{405,470}$ are measured at a single wavelength.

To explain this, we propose the following assumptions. Let's define the fluorescence spectrum excited by $\lambda_1 = 405$nm and $\lambda_2 = 470$nm as $f_{\lambda_1}(\lambda)$ and $f_{\lambda_1}(\lambda)$. As we can see in Fig.2 and Fig.3, the shape (or function w.r.t. wavelength) of the spectrum doesn't change with CO_2, and its amplitude scales proportionally with excitation intensity $A_{exc}^{\lambda_i} \propto I_{exc}^{\lambda_i}$ (also given the quantum yield) and, in the case of $\lambda_2 = 470$ nm, with CO_2 with some function $B = h(CO_2)$, meaning:

$$f_{\lambda_1}(\lambda) = A_{exc}^{\lambda_1} \cdot g_{\lambda_1}(\lambda)$$

$$f_{\lambda_2}(\lambda) = A_{exc}^{\lambda_2} \cdot B \cdot g_{\lambda_2}(\lambda)$$

so

$$I_{\lambda_1} = \int_{\lambda_{min}}^{\lambda_{max}} f_{\lambda_1}(\lambda)d\lambda = \int_{\lambda_{min}}^{\lambda_{max}} A_{exc}^{\lambda_1} \cdot g_{\lambda_1}(\lambda)d\lambda = A_{exc}^{\lambda_1} \cdot C_{\lambda_1}$$

$$I_{\lambda_2} = \int_{\lambda_{min}}^{\lambda_{max}} f_{\lambda_2}(\lambda)d\lambda = \int_{\lambda_{min}}^{\lambda_{max}} A_{exc}^{\lambda_2} \cdot B \cdot g_{\lambda_2}(\lambda)d\lambda = A_{exc}^{\lambda_2} \cdot B \cdot C_{\lambda_2}$$

with $A_{exc}^{\lambda_i}$ and $C_{\lambda_i} = \int_{\lambda_{min}}^{\lambda_{max}} g_{\lambda_i}(\lambda)d\lambda$ which do not depend on CO_2.

Therefore:

$$R = \frac{I_{\lambda_1}}{I_{\lambda_2}} = \frac{A_{exc}^{\lambda_1} \cdot C_{\lambda_1}}{A_{exc}^{\lambda_2} \cdot B \cdot C_{\lambda_2}}$$

and if the excitation source intensities, or their ratio ($A_{exc}^{\lambda_1}/A_{exc}^{\lambda_2} = const$) remain constant, and because the fluorescence spectra do not change shape, therefore the integrals C_{λ_i} do not

change, then the ratio changes proportionally to CO_2 through $B = h(CO_2)$, i.e., $R = K \cdot B^{-1}$ with $K = (A_{exc}^{\lambda_1} \cdot C_{\lambda_2})/(A_{exc}^{\lambda_2} \cdot C_{\lambda_2}) = constant$.

If we do not integrate but pick a single wavelength, say $\lambda_0 = 525nm$, then:

$$I_{\lambda_1}(\lambda_0) = A_{exc}^{\lambda_1} \cdot g_{\lambda_1}(\lambda_0)$$

$$I_{\lambda_2}(\lambda) = A_{exc}^{\lambda_2} \cdot B \cdot g_{\lambda_2}(\lambda_0)$$

and

$$R = \frac{I_{\lambda_1}}{I_{\lambda_2}} = \frac{A_{exc}^{\lambda_1} \cdot g_{\lambda_1}(\lambda_0)}{A_{exc}^{\lambda_2} \cdot B \cdot g_{\lambda_2}(\lambda_0)}$$

which is equivalent to R when we integrate the spectrum, where the constants C_{λ_i} are replaced by $\mathrm{g}_{\lambda_i}(\lambda_0)$.

Additionally, calculating the ratio R using the integral value yields a smoother function compared to calculating it using the intensities at a single wavelength, as the integral provides an averaging of the spectrum.

Performance of the calibration algorithms

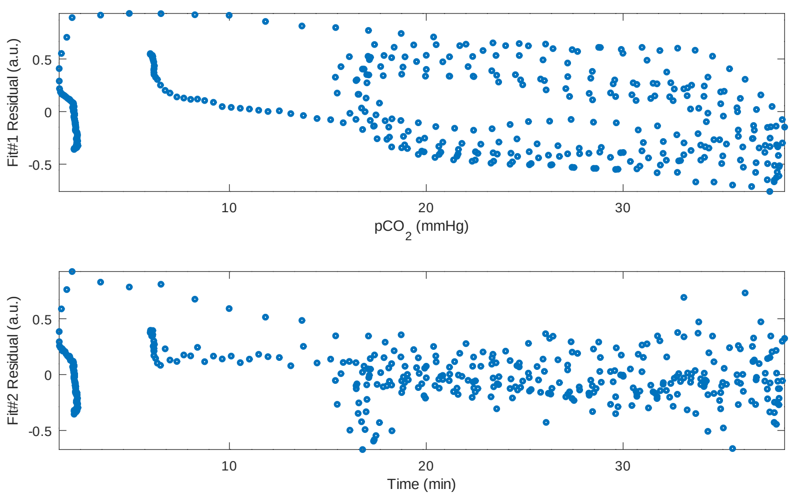

Figure S5: Residuals from (a) $Fit1$ and (b) $Fit2$. It can be clearly seen how $Fit2$ describes the data with higher fidelity, as the residual is randomly scattered, compared to $Fit1$, in which the trend of the data can still be detected.

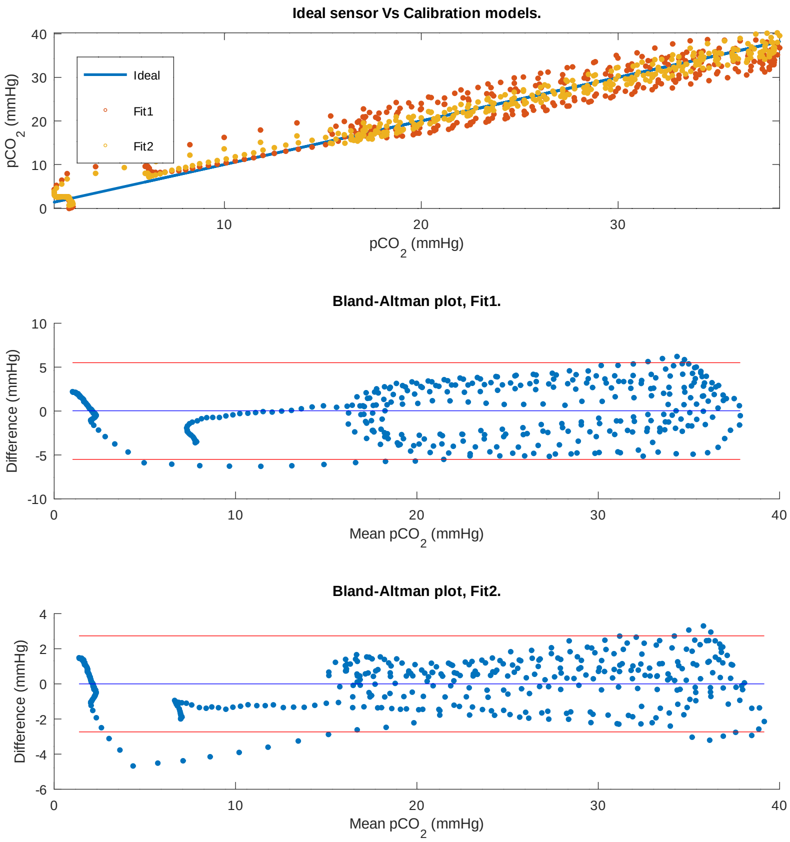

Figure S6: (a) Ideal Vs. calibration model sensor response. (b) Bland-Altman plot for $Fit1$. (c) Bland-Altman plot for $Fit2$. The standard deviation of $Fit2$ is roughly half of $Fit1$.

Linear calibration algorithm

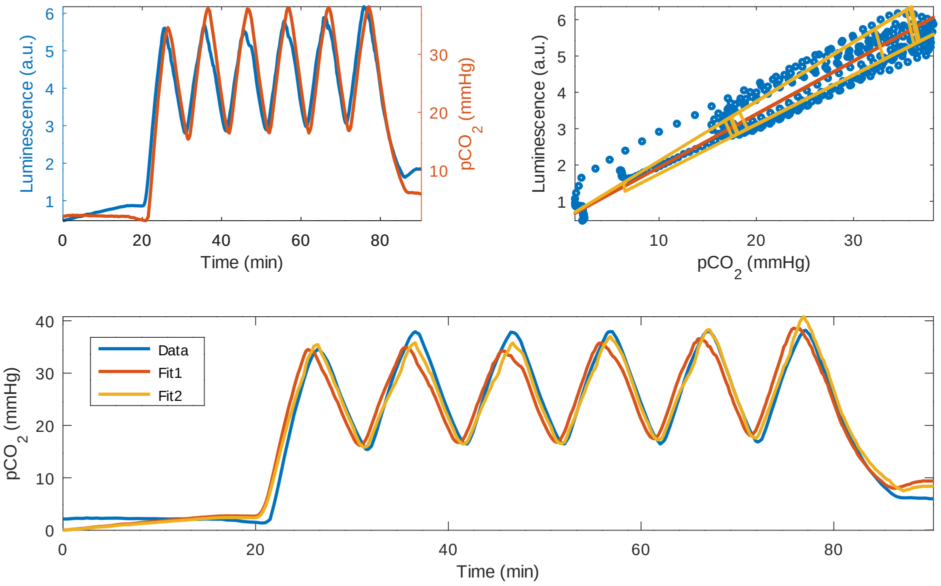

Figure S7: Fitting of a linear model to the luminescence ratio Vs. CO_2, as in Ref.,[1] which does not fully capture the curvature of the data.

References

(1) Zhu, Q.; Aller, R.C.; Fan, Y. A new ratiometric, planar fluorosensor for measuring high resolution, two-dimensional pCO2 distributions in marine sediments. *Marine Chemistry* **2006**, *101*, 40–53.
